# Does Pet Ownership in Infancy Lead to Asthma or Allergy at School Age? Pooled Analysis of Individual Participant Data from 11 European Birth Cohorts

**DOI:** 10.1371/journal.pone.0043214

**Published:** 2012-08-29

**Authors:** Karin C. Lødrup Carlsen, Stephanie Roll, Kai-Håkon Carlsen, Petter Mowinckel, Alet H. Wijga, Bert Brunekreef, Maties Torrent, Graham Roberts, S. Hasan Arshad, Inger Kull, Ursula Krämer, Andrea von Berg, Esben Eller, Arne Høst, Claudia Kuehni, Ben Spycher, Jordi Sunyer, Chih-Mei Chen, Andreas Reich, Anna Asarnoj, Carmen Puig, Olf Herbarth, Jestinah M. Mahachie John, Kristel Van Steen, Stefan N. Willich, Ulrich Wahn, Susanne Lau, Thomas Keil, GALEN WP 1.5 ‘Birth Cohorts’ working group, Magnus Wickman, Eva Hallner, Johan Alm, Catarina Almqvist, Göran Wennergren, Bernt Alm, Joachim Heinrich, Henriette A. Smit, Carel Thijs, Monique Mommers, Carsten Bindslev-Jensen, Susanne Halken, Maria Pia Fantini, Francesca Bravi, Daniela Porta, Francesco Forastiere, Adnan Custovic, Ruta Dubakiene, Jestinah Mahachie

**Affiliations:** Sachs' Children's Hospital; Institute of Environmental Medicine; Institute of Environmental Medicine; Dept. of Medical Epidemiology and Biostatistics, Karolinska Institutet, Stockholm, Sweden; Dept. of Pediatrics, Queen Silvia Children's Hospital, University of Gothenburg, Sweden; Dept. of Pediatrics, Queen Silvia Children's Hospital, University of Gothenburg, Sweden; Institute of Epidemiology, Helmholtz-Zentrum München, German Research Centre for Environmetal Health, Munich, Germany; Center for Prevention and Health Services Research, National Institute for Public Health and the Environment, Bilthoven, The Netherlands; School of Public Health and Primary Care, Maastricht University, Maastricht, The Netherlands; Dept. of Dermatology; Dept. of Pediatrics, Odense University Hospital, Denmark; Dept. of Public Health, Alma Mater Studiorum, University of Bologna, Italy; Dept. of Public Health, Alma Mater Studiorum, University of Bologna, Italy; Dipartimento di Epidemiologia ASL Rm E, Rome, Italy; Dipartimento di Epidemiologia ASL Rm E, Rome, Italy; Wythenshawe Hospital, University of Manchester, UK; Allergy Center, Vilnius University, Lithuania; Dept. of Oto-Rhino-Laryngology, University of Ghent, Belgium; 1 Department of Paediatrics, Oslo University Hospital, Ullevål, Norway; 2 Faculty of Medicine, University of Oslo, Oslo, Norway; 3 Institute for Social Medicine, Epidemiology and Health Economics, Charité - Universitätsmedizin Berlin, Berlin, Germany; 4 Voksentoppen, Oslo University Hospital, Rikshospitalet, Oslo, Norway; 5 Centre for Prevention and Health Services Research, National Institute for Public Health and the Environment, Bilthoven, The Netherlands; 6 Institute for Risk Assessment Sciences, Utrecht University, Utrecht, The Netherlands; 7 Julius Center for Health Sciences and Primary Care, University Medical Center, Utrecht, The Netherlands; 8 Ib-salut Menorca Health Area and Fundacio Caubet-Cimera, Menorca, Spain; 9 The David Hide Asthma and Allergy Research Centre, Isle of Wight and University of Southampton, Southampton, United Kingdom; 10 Institute of Environmental Medicine, Karolinska Institutet, Stockholm, Sweden; 11 Institut für Umweltmedizinische Forschung, University of Düsseldorf, Düsseldorf, Germany; 12 Department of Pediatrics, Marien-Hospital Wesel, Wesel, Germany; 13 Allergycenter, Department of Dermatology, Odense University Hospital, Odense, Denmark; 14 Department of Pediatrics, Odense University Hospital, Odense, Denmark; 15 Institute for Social and Preventive Medicine, University of Bern, Bern, Switzerland; 16 Environmental Epidemiology Research Centre and Institut Municipal d'Investigacio Medica, Barcelona, Spain; 17 Institute of Epidemiology, Helmholtz Zentrum München, German Research Centre for Environmental Health, Neuherberg, Germany; 18 Astrid Lindgrens' Children's Hospital, Karolinska University Hospital, Solna, Sweden; 19 Unitat recerca infancia i entorn, Institut Municipal d'Investigacio Medica - Hospital del Mar, Barcelona, Spain; 20 Faculty of Medicine, University of Leipzig and Department of Human Exposure Research and Epidemiology, UFZ - Centre for Environmental Research Leipzig, Leipzig, Germany; 21 Dept. of Oto-Rhino-Laryngology, University of Ghent, Ghent, Belgium; 22 Systems and Modeling Unit, Montefiore Institute, University of Liège, Liège, Belgium; 23 Bioinformatics and Modeling, GIGA-R, University of Liège, Liège, Belgium; 24 Dept. for Paediatric Pneumology and Immunology, Charité - Universitätsmedizin Berlin, Berlin, Germany; 25 Global Allergy and Asthma European Network (GA^2^LEN)-Work Package 1.5 ‘Birth Cohorts’; Hospital for Sick Children, Canada

## Abstract

**Objective:**

To examine the associations between pet keeping in early childhood and asthma and allergies in children aged 6–10 years.

**Design:**

Pooled analysis of individual participant data of 11 prospective European birth cohorts that recruited a total of over 22,000 children in the 1990s.

**Exposure definition:**

Ownership of only cats, dogs, birds, rodents, or cats/dogs combined during the first 2 years of life.

**Outcome definition:**

Current asthma (primary outcome), allergic asthma, allergic rhinitis and allergic sensitization during 6–10 years of age.

**Data synthesis:**

Three-step approach: (i) Common definition of outcome and exposure variables across cohorts; (ii) calculation of adjusted effect estimates for each cohort; (iii) pooling of effect estimates by using random effects meta-analysis models.

**Results:**

We found no association between furry and feathered pet keeping early in life and asthma in school age. For example, the odds ratio for asthma comparing cat ownership with “no pets” (10 studies, 11489 participants) was 1.00 (95% confidence interval 0.78 to 1.28) (I^2^ = 9%; p = 0.36). The odds ratio for asthma comparing dog ownership with “no pets” (9 studies, 11433 participants) was 0.77 (0.58 to 1.03) (I^2^ = 0%, p = 0.89). Owning both cat(s) and dog(s) compared to “no pets” resulted in an odds ratio of 1.04 (0.59 to 1.84) (I^2^ = 33%, p = 0.18). Similarly, for allergic asthma and for allergic rhinitis we did not find associations regarding any type of pet ownership early in life. However, we found some evidence for an association between ownership of furry pets during the first 2 years of life and reduced likelihood of becoming sensitized to aero-allergens.

**Conclusions:**

Pet ownership in early life did not appear to either increase or reduce the risk of asthma or allergic rhinitis symptoms in children aged 6–10. Advice from health care practitioners to avoid or to specifically acquire pets for primary prevention of asthma or allergic rhinitis in children should not be given.

## Introduction

The causes of the worldwide asthma and allergy epidemic over recent decades remain uncertain. Environmental and lifestyle factors, possibly interacting with genetic variants, may play a role however clear evidence for a predominant risk factor is lacking. Pet exposure as a common indoor environmental exposure particularly in families with young children has been of increasing public health concern with regard to recommendations for primary prevention of respiratory and allergic disease. Considerable controversy exists as to whether particularly cat and dog exposure may be a risk or even a protective factor for developing asthma, allergic symptoms or allergic sensitization [1]–[24]. The conferred risks of pet exposure may be limited to individuals with allergic parents [25]–[27].

Previous results have come predominantly from cross-sectional studies and may therefore be skewed due to recall bias with regards to pet keeping [7], and early symptoms [28]; in addition, pet avoidance behaviour may distort the associations between pets and allergic diseases [15], [29], [30]. The heterogeneity of results might also be explained by differences in exposure classifications without “clean” categories of single pets and differences in the prevalence of pets in the community [31]. Furthermore, the climate may influence indoor versus outdoor pet keeping and its association with allergic outcomes [3], [32].

Primary care practitioners are uncertain about respiratory health risks or benefits of furry pet ownership particularly in early childhood and what advice to give to parents. The objective of this study was to improve the evidence on the primary prevention of asthma and allergies in relation to pet keeping in early life, using data from a large data base of European birth cohort studies. The primary aim was to determine whether pet keeping in the first two years of life was associated with asthma in school-aged children (age 6 to 10 years). Secondarily, we aimed to assess whether pet-keeping was associated with other allergic diseases (allergic or non-allergic asthma, allergic sensitization or allergic rhinitis).

## Methods

### Design and included birth cohort studies

As part of the Global Allergy and Asthma European Network (GA^2^LEN, www.ga2len.net) all population-based European birth cohort studies with a special focus on asthma and allergy were identified, contacted and their methods described and compared [33], [34].

For the present combined data analyses, three inclusion criteria were defined: (i) European population-based observational birth cohort studies focusing on allergy and asthma (with ethical approval from local review boards); (ii) recruitment of subjects in pregnancy, at birth or during the first year of life; (iii) at least 1 prospective assessment during 6–10 years of age (early school age); (iv) data on pet ownership assessed prospectively during the first 2 years. To avoid recall bias about early childhood exposures, cross-sectional studies of school-children were not considered. For each included study the raw individual level participant data was available for data analysis.

### Ethics statement

This meta-analysis was conducted according to the principles stated in the Declaration of Helsinki. All included birth cohort studies were approved by their local Institutional Review Boards and all participants' parents provided written informed consent. The Institutional Review Boards were for MAS: Ethical Review Board Charité – Universitätsmedizin Berlin, Berlin (Germany); BAMSE: Regional Ethical Review Board, Karolinska Institutet, Stockholm (Sweden); ECA: The regional committee for medical and health profession research ethics, South-East, (Norway); PIAMA-NHS: Ethical Review Boards Utrecht CCMO P04.0071C, Rotterdam MEC 2004-152, Groningen M 4.019912 (The Netherlands); LISA: Ethics committees of the Bavarian General Medical Council, the University of Leipzig, and the Medical Council of North-Rhine-Westphalia (Germany); GINI-B: Ethics committees of the Bavarian General Medical Council, the University of Leipzig, and the Medical Council of North-Rhine-Westphalia (Germany); ARC: The Regional Scientific Ethical Committee for Southern Denmark (Denmark); AMICS-Barcelona: Clinical Research Ethical Committee of the Parc de Salut Mar, IMIM, Barcelona (Spain); AMICS-Menorca: Comite etic d'investigacio clinica de les Illes Balears (Spain); Leicester: Leicestershire, Northamptonshire and Rutland Research Ethics Committees 1 and 2 (UK); Isle of Wight: lsle of Wight, Portsmouth & SE Hants HA Local Research Ethics Committee (UK).

### Definition of primary outcome

Since current “wheeze” is not very specific for asthma [35], we chose the primary outcome to be “current asthma” for the last available follow-up during 6–10 years defined as satisfying at least 2 out of 3 parent-reported conditions (from self-report questionnaires or interviews): (i) doctor-diagnosed asthma ever; (ii) asthma symptoms/wheezing (last 12 months) according to the International Study of Asthma and Allergy in Childhood (ISAAC) core questions [36]; (iii) using asthma medication (last 12 months) [35]. For two studies (DARC, ECA) the study physician's asthma diagnosis was used.

### Definition of secondary outcomes

“Allergic asthma” was defined as the presence of the primary outcome “asthma” and a positive serum specific immunoglobulin E (s-IgE)>0.35 kU/l to (i) any aero- and/or food allergen. Further definitions of allergic asthma were specified as asthma with a positive s-IgE to: (ii) any aero-allergen (in- or outdoor); (iii) cat allergen; (iv) dog allergen. “Non-allergic asthma” was defined as the presence of “asthma” without sensitization to any tested aero-/food allergen (s-IgE≤0.35 kU/l). The reference groups were non-asthmatic children without allergic sensitization.

“Allergic sensitization” regardless of symptoms was defined as a positive s-IgE test >0.35 kU/l for the following categories: cat, dog, any indoor, any outdoor, any aero-, and any aero-/food allergen.

“Allergic rhinitis” included parent-reported symptoms during the last 12 months (ISAAC core questions: sneezing, runny or blocked nose without a cold or flu) plus s-IgE>0.35 kU/l against at least 1 aero-allergen.

### Definition of household pet keeping

Based on parent-completed questionnaires or interviews between the children's birth (or during pregnancy) and second birthday, we defined 6 pet ownership categories: (i) cat(s) only; (ii) dog(s) only; (iii) cat(s) and dog(s) only; (iv) rodent(s) only; (v) bird(s) only; (vi) and no furry or feathered pets (“no pets”) as the reference category. Six percent of families could not be classified into one of the categories above because they had a combination of different types of pets and were thus excluded from the analyses. Information on pet contact outside the home or outdoor pet keeping was not available in most cohorts. Other pets such as reptiles or amphibians were not considered.

Our primary aim was to examine the effect of pet ownership at any time between birth and the 2^nd^ birthday. In addition, to evaluate whether the timing of pet ownership is relevant, we examined different exposure periods: at time of birth; between birth and 1^st^ birthday, and between 1^st^ and 2^nd^ birthdays.

### Definition of possible confounding factors

Eleven variables, if available, collected by parental questionnaires or interviews, were considered as possible confounders in the adjusted analyses of the individual birth cohorts: 1. family history (parents and siblings) of asthma and/or allergic rhinitis (yes versus no); 2. family history of pet allergy (yes versus no); 3. maternal smoking during pregnancy (yes versus no); 4. postnatal maternal smoking from after birth to last follow-up between 6 to 10 years of age (‘regular smoker’ and ‘irregular smoker’ versus ‘never smoke’ as reference category); 5. educational level of parents at birth of child (by tertile according to school years as proxy for socio-economic status); 6. one or more older siblings (yes versus no); 7. home/apartment with convenient ground access (ground or 1st floor versus 2nd floor or higher); 8. crowding at home (number of persons per square meter or room; in quintiles, with the lowest quintile as reference category); 9. gender (boys versus girls); 10. breast feeding duration (in months); 11. doctor's diagnosed eczema any time between birth and 2 years (yes versus no).

### Statistical analyses

For each cohort, a multivariable logistic regression analysis was used to calculate the adjusted odds ratio (OR) and 95% confidence intervals (CI) to estimate the effect of pet exposure in the first 2 years on the primary (current asthma) and secondary outcomes at age 6 to 10 years. Adjustment was performed for 7 potential confounders that were available for all studies (these were factors 1, 4–6, and 9–11 as listed above) and in addition, for all factors available for the respective cohort. Furthermore, we performed sensitivity analyses using (i) only the 7 potential confounders available for all studies, and (ii) using a propensity score approach for adjustment [37], [38]. For the latter, all available covariates as listed above (except gender of the child) were used for each study separately to estimate scores indicating the propensity of pet ownership for each participant using logistic regression analysis; subsequently these propensity scores plus gender were used as adjustment variables for modelling pet ownership and outcomes. For the primary outcome, we additionally analyzed possible two-way interactions (effect modification) between pet exposure and (i) parental allergy status, (ii) smoking in pregnancy and (iii) postnatal maternal smoking.

The combining of results from all cohorts was done by random-effect meta-analyses with the inverse-variance method, based on the assumption that the associations in the different cohorts are not identical, estimating the average of the associations [39], [40]. As further sensitivity analyses for the primary outcome, we calculated fixed-effect meta-analyses, where it is assumed that the association is the same across all cohorts [39].

In subgroup analyses, we assessed the associations for the following groups: (i) parents with and (ii) without asthma or allergic rhinitis ever; (iii) parents with and (iv) without pet allergies ever; (v) parents with asthma and/or allergic rhinitis, but without pet allergies; and (vi) parents without any allergies. Furthermore, we analyzed studies with high and those with low prevalence of pet ownership separately, and compared cohorts from major climatic regions in Europe (Nordic, Maritime, Central, and South).

In all analyses, a level of 0.05 was considered as statistically significant, without adjustment for multiple testing. Heterogeneity among the studies was tested using chi-squared Q-statistic and I^2^. We performed meta-analyses with Review Manager version 5.0 (German Cochrane Centre, Freiburg, Germany) and all other analyses with SAS version 9.1 (SAS Institute, Cary, NC, USA).

## Results

11 European studies, including the largest and oldest birth cohorts that were specifically designed to examine asthma and allergies, expressed interest and were included in the combined analyses. The recruitment of newborns and their families took place from 1989 (Isle of Wight, UK) to 1998 (DARC, Denmark and Leicester, UK) (Table 1). During age 6 to 10 years most cohorts achieved a follow-up rate of over 75%, this being the highest in the Isle of Wight and the three Scandinavian cohorts (Table 1).

**Table 1:** Birth cohort acronym, study setting, year of recruitment, number of children recruited, and pet ownership during the first 2 years of life in 11 European birth cohorts (sorted from north to south).

Birth cohort acronym	Study setting/first year of recruitment	Children initially recruited, N	Pet ownership at age 0–2 y					
			%					
			(n/N)					
			Cat(s) only^1^	Dog(s) only^1^	Cat(s) and dog(s) only^1^	Bird(s) only^1^	Rodent(s) only^1^	No furry or feathered pets
ECA	Oslo	3754	7.9	8.8	0.9	4.3	1.5	76.6
	Norway, 1992	(1877)^2^	(222/2810)	(247/2810)	(24/2810)	(122/2810)	(42/2810)	(2153/2810)
BAMSE	Stockholm	4089	9.6	4.4	1.5	2.4	1.6	80.6
	Sweden, 1994		(355/3719)	(163/3719)	(54/3719)	(89/3719)	(60/3719)	(2998/3719)
DARC	Odense	562	16.2	11.0	4.0	3.6	2.3	62.9
	Denmark, 1998		(72/445)	(49/445)	(18/445)	(16/445)	(10/445)	(280/445)
Leicester 1998	Leicester	566^3^	16.4	16.4	4.4	1.5	6.5	54.8
	UK, 1998		(86/524)	(86/524)	(23/524)	(8/524)	(34/524)	(287/524)
Isle of Wight	Isle of Wight	1456	25.5	18.5	11.5	3.5	2.5	38.5
	UK, 1989		(274/1074)	(199/1074)	(123/1074)	(38/1074)	(27/1074)	(413/1074)
PIAMA-NHS	Multicenter	3291	27.8	8.7	5.7	6.1	6.2	45.5
	The Netherlands, 1996		(729/2620)	(228/2620)	(148/2620)	(160/2620)	(163/2620)	(1192/2620)
MAS	Multicenter	1314	11.6	6.1	1.6	8.5	3.4	68.8
	Germany, 1990		(108/933)	(57/933)	(15/933)	(79/933)	(32/933)	(642/933)
LISA	Multicenter	3097	9.6	6.1	1.5	4.7	5.4	72.7
	Germany, 1997		(232/2406)	(147/2406)	(37/2406)	(112/2406)	(129/2406)	(1749/2406)
GINI-B	Multicenter	3739	6.8	7.2	1.9	4.8	4.3	75.0
	Germany, 1996		(162/2377)	(172/2377)	(44/2377)	(113/2377)	(103/2377)	(1783/2377)
AMICS-Barcelona	Barcelona	487	6.2	9.8	1.0	21.8	4.2	57.0
	Spain, 1996		(12/193)	(19/193)	(2/193)	(42/193)	(8/193)	(110/193)
AMICS-Menorca	Menorca	485	4.3	18.3	6.5	18.1	2.0	50.8
	Spain, 1997		(17/398)	(73/398)	(26/398)	(72/398)	(8/398)	(202/398)
**Total**		**22840**	**13.0**	**8.2**	**2.9**	**4.9**	**3.5**	**67.5**
			**(2269/17499)**	**(1440/17499)**	**(514/17499)**	**(851/17499)**	**(616/17499)**	**(11809/17499)**

ECA: Environment and Childhood Asthma Study. BAMSE: Children, Allergy, Milieu, Stockholm, Epidemiological Survey. DARC: Danish Allergy Research Council Study. PIAMA-NHS: The Prevention and Incidence of Asthma and Mite Allergy – Natural History Study. MAS: Multi-center Allergy Study. LISA: Lifestyle-related Factors on the Immune System and Development of Allergies in Childhood Study. GINI-B: German Infant Nutritional Intervention Study (observational part). AMICS: Asthma Multicentre Infant Cohort Study.

1 and no other furry or feathered pet.

2 3754 initially recruited and followed-up until 2 years; for ECA: 1877 invited for longer follow-up.

3 More children are part of the Leicester 1998 cohort, but for the present meta-analyses on early pet ownership we included only children who were recruited during the 1^st^ year of life.

### Pet ownership

Pet ownership ranged from around 60% (Isle of Wight, UK) to around 20% (BAMSE, Stockholm, Sweden), only cat ownership from 28% (Dutch PIAMA-NHS) to 4% (Menorca, Spain), and only dog ownership from 18% (the 2 islands Menorca and Isle of Wight) to 4% (BAMSE) (Table 1). Keeping both cat(s) and dog(s) but no other pets was particularly common on the Isle of Wight (UK), keeping birds only in the 2 Spanish cohorts, and keeping rodents only in Leicester, UK, and the Dutch PIAMA-NHS cohort, respectively. Data to define the pet ownership categories was available for 40% (Menorca, Spain) to 93% (Leicester, UK).

### Primary endpoint

**Figure 1:**
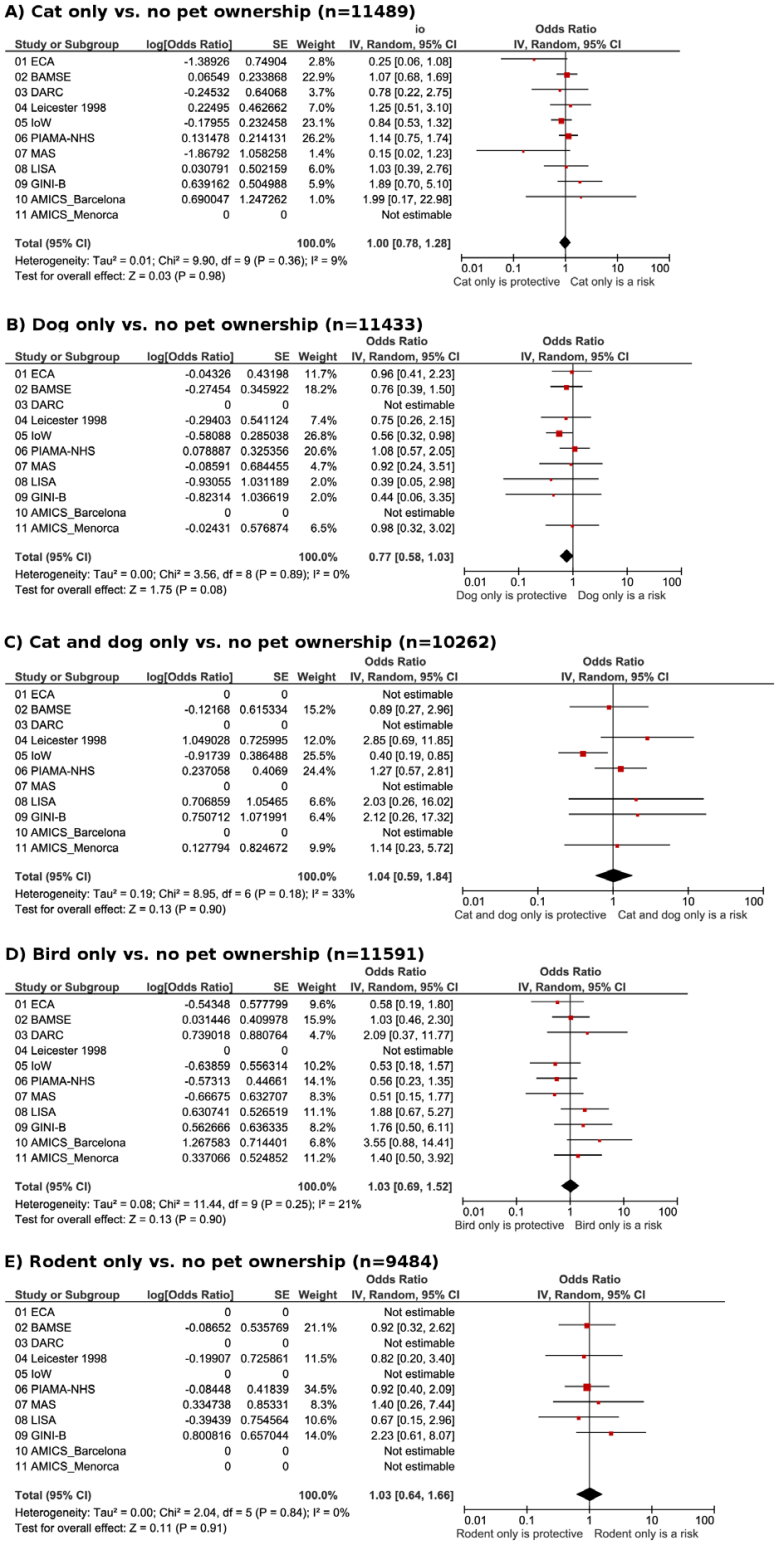
Current asthma. Meta-analyses of the adjusted odds ratios of **asthma** at 6–10 years of age and pet ownership in the first 2 years of life for: A), cat only vs. no pets; B), dog only vs. no pets; C) cat and dog only vs. no pets; D) bird only vs. no pets; E) rodents only vs. no pets.

The prevalence of current asthma at 6–10 years ranged from 2.9%–18.7% (Table 2). There were no significant associations between any type of pet ownership during the first 2 years and asthma during 6–10 years in the adjusted estimates of the main meta-analyses or in any of the individual cohorts (Figure 1). The meta-analysis odds ratio (OR) for asthma when owning a cat was 1.00 (95% confidence interval 0.78–1.28) and 0.77 (0.58–1.03) when owning a dog. Owning both cat and dog resulted in an OR for asthma of 1.04 (0.59–1.84). The OR of bird ownership was 1.03 (0.69–1.52), and 1.03 (0.64–1.66) for rodents. Heterogeneity across the cohorts was not significant.

**Table 2:** Prevalence of current asthma, allergic asthma (sensitized to ≥1 aero-allergen), allergic rhinitis (sensitized to ≥1 aero-allergen) and allergic sensitization (≥1 aero-allergen >0.35 kU/L) at last follow-up assessment between 6 to 10 years in 11 European birth cohorts.

Birth cohort, country (sorted from north to south)	Age of children at follow-up (years)	Follow-up rate	Asthma	Allergic asthma	Allergic rhinitis	Allergic sensitisation
			%	%	%	%
			(n/N)	(n/N)	(n/N)	(n/N)
ECA	10	84%	11.9	7.6	13.9	33.2
Norway			(120/1010)	(73/963)	(135/972)	(325/979)
BAMSE	8–9	84%	9.3	5.2	8.0	26.0
Sweden			(308/3330)	(165/3187)	(255/3202)	(637/2451)
						
DARC	6	81%	7.7	4.3^1^	4.1^1^	35.9^1^
Denmark			(35/457)	(19/441)	(18/441)	(168/468)
Leicester 1998	6	57%	18.7	n.a.	n.a.	n.a.
UK			(66/353)			
						
Isle of Wight	10	94%	16.3	8.8	11.9	33.0
UK			(223/1370)	(110/1257)	(142/1196)	(314/952)
PIAMA-NHS	8	83%	7.1	2.7	5.5	29.7
The Netherlands			(194/2720)	(70/2596)	(131/2374)	(383/1289)
MAS	10	58%	11.2	9.1	20.0	48.1
Germany			(68/606)	(54/592)	(147/735)	(343/713)
LISA	6	71%	3.0	1.2	5.5	26.7
Germany			(66/2185)	(25/2144)	(109/1988)	(318/1193)
GINI-B	6	59%	2.9	1.3	4.5	27.2
Germany			(64/2179)	(28/2143)	(90/2020)	(257/945)
AMICS-Barcelona	6	64%	12.5	3.9^1^	5.8^1^	18.9^1^
Spain			(39/312)	(11/284)	(15/259)	(54/286)
AMICS-Menorca	6	94%	7.9	3.0^1^	0.5^1^	12.3^1^
Spain			(36/458)	(13/435)	(2/438)	(43/349)
**Total**			**8.1**	**4.0**	**7.7**	**29.5**
			**(1219/14980)**	**(568/14042)**	**(1044/13625)**	**(2842/9625)**

n.a. = not assessed.

1 in DARC, AMICS-Barcelona and AMICS-Menorca, sensitization data were only available for the age of 4 years.

Main results were similar when analyzing shorter pet exposure time periods (e.g. around birth or during first 12 months) or in sensitivity analyses using a propensity score to control for potential confounding. Also, meta-analyses in subgroups showed no significant association of pet ownership and asthma among parents with or among those without asthma/allergies, in cohorts with only high or those with only low pet prevalence, or in subgroups of cohorts from 4 major climatic regions in Europe.

No significant associations that would suggest effect modification were found when we analyzed two-way interactions between pet exposure and parental allergies, maternal prenatal smoking, or postnatal maternal smoking. Results were similar for fixed compared with random effect meta-analyses.

### Secondary endpoints

#### Allergic asthma

**Figure 2:**
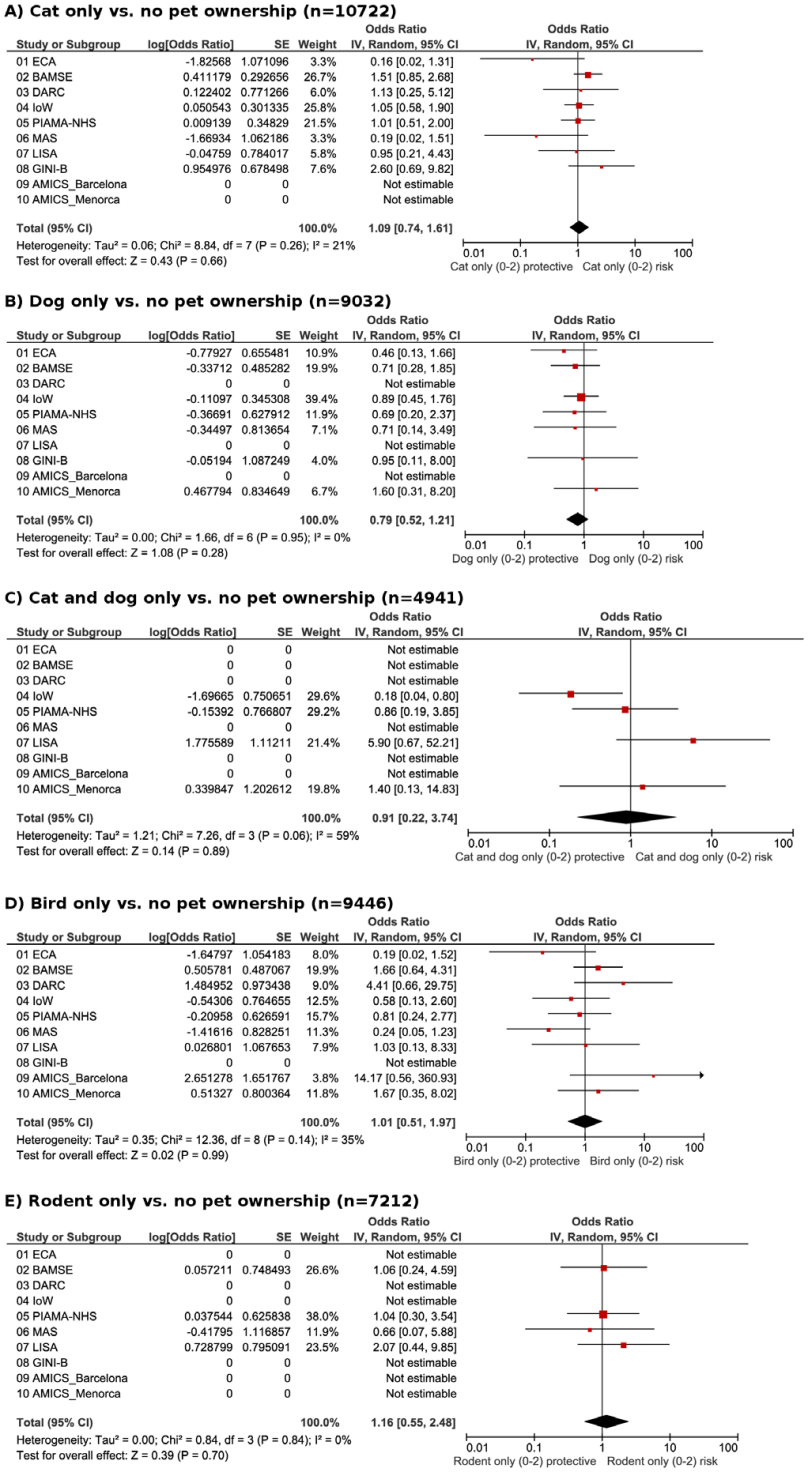
Allergic asthma. Meta-analyses of the adjusted odds of **allergic asthma** (sensitized to at least 1 aero-allergen; secondary endpoint) in early school age and ownership of pets in the first 2 years of life for: A), cat only vs. no pets; B), dog only vs. no pets; C) cat and dog only vs. no pets; D) bird only vs. no pets; E) rodents only vs. no pets.

The overall prevalence of allergic asthma (defined as current asthma and sensitization to ≥1 aero-allergen) in early school age was 4.0%, ranging from 1.2% at six years to 9.1% at 10 years (Table 2). Pet ownership was not associated with asthma in combination with sensitization to ≥1 aero-allergen (Figure 2), to ≥1 indoor-, to ≥1 outdoor, or to ≥1 aero- or food allergen in the meta-analyses of all cohorts (data not shown). Based on results from only 3 cohorts with data available, owning a dog was not associated with asthma in combination with sensitization to dog (OR 1.14, 95% CI 0.57–2.28) or to cat. However, owning cats increased the odds of having asthma combined with sensitization to dog (OR 2.59, 95% CI 1.49–4.49) and to cat (OR 1.91, 95% CI 1.16–3.12).

#### Non-allergic asthma

Asthma without sensitization to any aero- or food allergen (“non-allergic asthma”) was not significantly associated with cat (OR 0.99, 0.51–1.94), dog (OR 1.35, 0.71–2.57), bird (OR 1.80, 0.80–4.04) or rodent ownership (OR 1.70, 0.53–5.44) in the meta-analyses of all cohorts. Results from the only 2 cohorts with sufficient data regarding both cat and dog ownership (BAMSE and PIAMA-NHS) showed that owning both cat(s) and dog(s) increased the odds of non-allergic asthma (OR 3.66, 1.50–8.93).

#### Allergic sensitization and rhinitis

**Figure 3:**
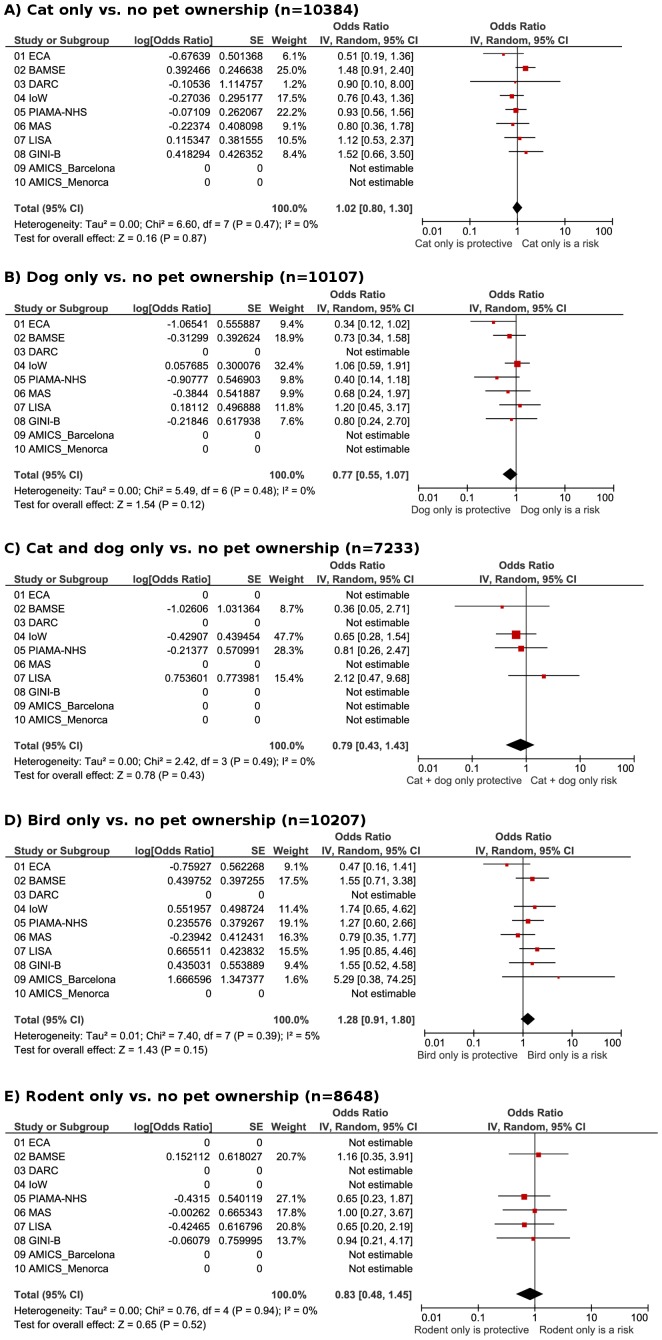
Allergic sensitization. Meta-analyses of the adjusted odds of **allergic sensitization** (sensitized to at least 1 aero-allergen) in early school age and pet ownership in the first 2 years of life for: A), cat only vs. no pets; B), dog only vs. no pets; C) cat and dog only vs. no pets; D) bird only vs. no pets; E) rodents only vs. no pets. (There were no IgE data available for Leicester 1998 cohort.)

**Figure 4:**
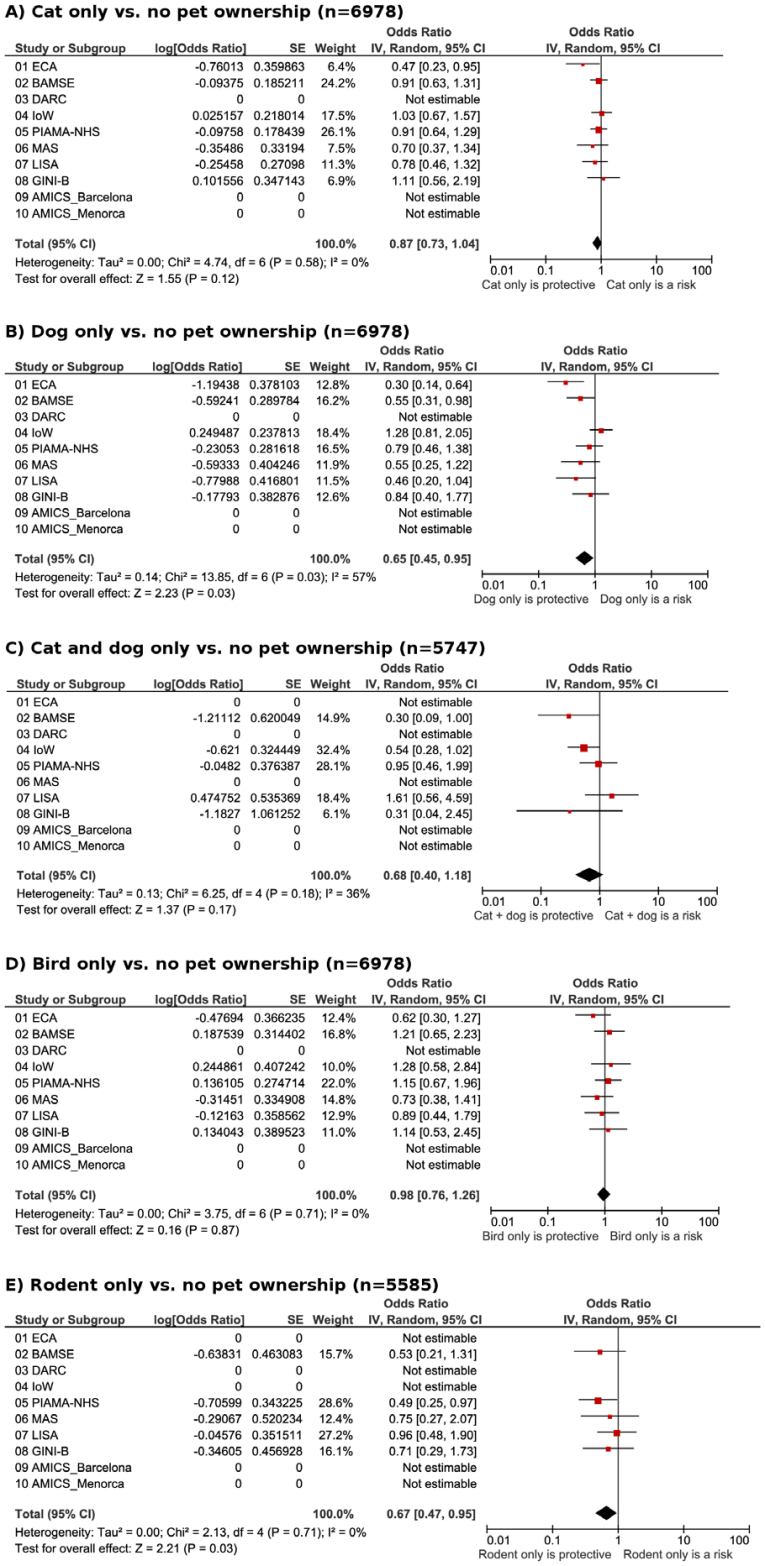
Allergic rhinitis. Meta-analyses of the adjusted odds of **allergic rhinitis** (sensitized to at least 1 aero-allergen; secondary endpoint) in early school age and pet ownership in the first 2 years of life for: A), cat only vs. no pets; B), dog only vs. no pets; C) cat and dog only vs. no pets; D) bird only vs. no pets; E) rodents only vs. no pets.

The prevalence of sensitization to ≥1 aero-allergen ranged from 26%–33% during 6–10 years (Table 2). Having dogs or rodents during the first 2 years significantly reduced the odds of sensitization to ≥1 aero-allergen (OR 0.65, 0.45–0.95 for dog; OR 0.67, 0.47–0.95 for rodent; Figure 3). Cat ownership showed a similar trend (OR 0.87, 0.73–1.04). The prevalence of parent-reported rhinitis during 6–10 years plus sensitization to ≥1 aero-allergen was 7.7% (Table 2). Allergic rhinitis was not associated with any pet ownership (Figure 4).

Insufficient data for the definition of the primary endpoint ranged from 5% (AMICS-Menorca) to 54% (MAS) of the participants; from 10% (AMICS-Menorca) to 55% (MAS) for defining allergic asthma; 10% (AMICS-Menorca) to 48% (ECA) for allergic rhinitis, and 17% (DARC) to 75% (GINI-B) for the definition of allergic sensitization.

## Discussion

### Principal findings

Our meta-analyses showed that ownership of single types of furry pets or birds in the first 2 years of life neither increased nor decreased the risk of asthma, non-allergic asthma (not sensitized to any aero- or food-allergen), allergic asthma or allergic rhinitis (both included sensitization to at least 1 aero-allergen) in school-aged children. However, living with furry pets in the first 2 years appeared to reduce the likelihood of becoming sensitized to aero-allergens in early school-age regardless of respiratory symptoms.

### Comparison with other studies

An older meta-analysis, mainly with cross-sectional studies from the 1990s, showed a slightly increased risk of asthma or wheezing for children >6 years in relation to any pet exposure, but did not analyze different types of pets [41]. A more recent meta-analysis of 9 cohort studies (including children of all ages) showed a protective effect for asthma related to cat exposure [42]. Both previous meta-analyses were based on published risk estimates with the disadvantage that exposure, potential confounders and outcome could not be harmonized across the included studies compared to our analyses using individual raw data from 11 birth cohort studies with long-term prospective assessments.

The reduced sensitization to aero-allergens related to furry pet ownership is consistent with similar findings in several previous studies, particularly for dogs [7], [21], [22], [24], [43]. We found that rodent ownership showed this protective effect too, and that dog ownership was associated with reduced risk of sensitization to common food allergens (data not shown).

### Strengths of present analyses

Our approach was different than a previous meta-analysis on this topic because we were able to collect, harmonize, and combine the individual participant data from 11 birth cohorts instead of using published risk estimates based on heterogeneous outcome and exposure definitions and age groups [42].

The large sample allowed the definition of mutually exclusive pet exposure categories: ownership of “only cat(s)”, “only dog(s)”, “only rodent(s)”, and “only bird(s)”. This is another unique feature of our collaborative study compared to previous studies, which did not separate potential effects of “clean” pet exposure categories. Furthermore, analyzing the time at birth, the first and the second year of life separately, the results were very similar compared with the whole period of the first 2 years. This suggests that our results are robust, and do not point towards a narrow post natal period with increased susceptibility to pet exposure in the home.

For the outcome definition, previous studies used single variables such as parent-reported wheezing or doctor's-diagnosed asthma, which may have over- or underestimated the real prevalence of asthma. To avoid a potential over-estimation of asthma prevalence we used a more stringent definition for the primary outcome asthma based on at least 2 out of the 3 conditions parent-reported wheezing, doctor's-diagnosed asthma and asthma medication [35]. Also, pet ownership was not assessed in relationship to severity of asthma since our aim was to investigate the possible role of pets in primary prevention of asthma. Our definition of allergic rhinitis was not only based on typical symptoms but also included detection of serum IgE.

A limitation of previous studies may have been the lack of sufficient adjustment for potential confounding. The size of our sample had enough statistical power to take into account potential confounders including family, social and domestic factors in most cohorts [31].

Although the birth cohorts come from different climatic European regions, include children born in different years (between 1989–1998), have urban and rural/island study settings, different prevalences of allergies and patterns of pets, the statistical tests for heterogeneity were rarely significant, which strengthens the findings and generalizability of our analyses.

### Possible limitations

Avoidance behavior in families with allergies could be an explanation for the “protective effect” of pet keeping seen in some previous studies (reverse causation) [7]; however reasons for avoiding pets were not assessed in most birth cohorts. We addressed the issue of avoidance behavior due to parental allergies to some extent by running meta-analyses in several subgroups. The results were very similar among children from parents *without* allergies (asthma, allergic rhinitis, pet allergy) compared to children from parents *with* allergies. Furthermore, keeping certain types of pets may be associated with different life-styles that we were unable to account for in the present meta-analyses.

Another possible limitation of our analyses is that for some birth cohorts the outcome was only available for 6 years of follow-up (at this age asthma may not have been fully developed in some subjects), whereas for others we could include the 8 and 10 year follow-up data. Comparing the effect estimates of the individual birth cohorts, we did not find that cohorts with a 6 year follow-up differed from the older cohorts; instead we found rather homogeneous results across the cohorts.

Some cohorts assessed more potential confounding variables than others. However, when we repeated our analyses with only those confounders that were assessed in all studies our results did not change considerably. Since most cohorts did not ask for the number of pets at home, we could not examine the possibility of a dose-response relationship of pet keeping.

A risk of participation bias in each included study could be present and it could be different for each study (e.g. due to regional differences in disease awareness or in recruitment strategies). However, while this might influence the observed prevalences for allergic diseases, this should less influence any association between pet ownership and allergic disease. On the other hand, each cohort had different numbers of observations available to define the primary and secondary outcomes, and some kind of selection bias cannot be excluded.

Assessing total exposure to pet allergens in early life was outside the aim and scope of the present study. Furthermore, total allergen exposure, which is virtually impossible to measure, would not influence the scientific evidence for giving advice on pet keeping or not.

When interpreting the results, the reliance upon parents' questionnaire data should be kept in mind. It should be noted, however, that standardized ISAAC questions were used to assess allergic symptoms and diseases. In addition, the quality of data might not be equal across the included studies due to data collection timing and methods.

We examined various secondary endpoints while performing over a hundred additional explorative analyses of the whole dataset and of subgroups. Some of these subgroups included only 2 or 3 birth cohorts if these were the only ones with sufficient exposure and/or outcome data. Almost all analyses showed no associations between exposure and secondary outcomes with a few exceptions, e.g. a positive association between cat ownership and asthma in combination with sensitization to dog or cat allergens, however dog ownership was not associated with asthma in combination with dog or cat allergens. Another positive association was found between ownership of both cat(s) and dog(s) and non-allergic asthma; however cat and dog ownership alone was not associated with non-allergic asthma. Since we did not correct for multiple testing, the statistically significant results in some of the subgroup analyses should be interpreted cautiously and as results of explorative analyses keeping in mind the possibility of false positive findings.

## Conclusions

This pooled analysis of individual participant data from 11 European birth cohorts found no association between ownership of single types of furry and feathered pets in the first 2 years of life and asthma or allergic rhinitis in school children aged 6–10. For primary prevention of asthma and allergy, we found no evidence for health care practitioners to give parents specific advice on avoiding or acquiring pets in early childhood. To evaluate the effect of pet keeping in early childhood on e.g. developing eczema, further pooled birth cohort data analyses are needed rather than single birth cohort analyses.
